# Different Stability-Indicating Chromatographic Techniques for the Determination of Netobimin

**DOI:** 10.1155/2012/754650

**Published:** 2012-02-16

**Authors:** Nesrin K. Ramadan, Afaf O. Mohamed, Sara E. Shawky, Maissa Y. Salem

**Affiliations:** ^1^Analytical Chemistry Department, Faculty of Pharmacy, Cairo University, Kasr El-Aini Street, Giza 11562, Egypt; ^2^Analytical Chemistry Department, National Organization for Drug Control and Research (NODCAR), 6 Abu Hazem Street, Pyramids Avenue, P.O. Box 29, 35521, Egypt; ^3^Department of Pharmaceutical Chemistry, Faculty of Pharmacy, Umm Al-Qura University, 13174 Makkah, Saudi Arabia

## Abstract

Two simple, accurate, and sensitive methods were developed for the determination of netobimin in the presence of its degradation product. Method (A) was an HPLC method, performed on C18 column using acetonitrile/methanol/0.01 M potassium dihydrogen phosphate (56 : 14 : 30 by volume) as a mobile phase with a flow rate of 0.5 mL/min. Detection was performed at 254 nm. Method (B) was a TLC method, using silica gel 60 F_254_ plates; the optimized mobile phase was toluene/methanol/chloroform/ammonium hydroxide (5 : 4 : 6 : 0.1 by volume). The spots were scanned densitometrically at 346 nm. Linearity ranges were 1–10 *μ*g/mL for method (A) and 0.5–5 *μ*g/band for method (B), and the mean percentage recoveries were 99.3 ± 0.7% and 99.7 ± 0.7% for methods (A) and (B), respectively. The proposed methods were found to be specific for netobimin in the presence of up to 90% of its degradation product. Statistical comparison between the results obtained by these methods and the manufacturer method was done, and no significance difference was obtained.

## 1. Introduction

**Scheme 1:**
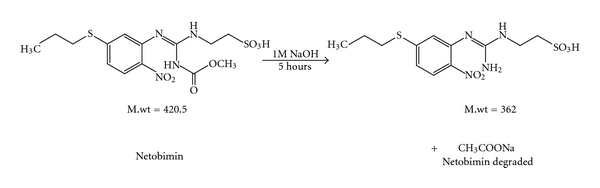
The degradation pathway of netobimin.

Netobimin, 2-{3-meth-oxycarbonyl-2-[2-nitro-5-(propylthio)phenyl]guanidi-one} ethanesulphonic acid, is an anthelmintic drug used in veterinary medicine, Scheme 1 [1]. It is a probenzimidazole with primary antinematodal activity at a dosage of 7.5 mg/kg, and at elevated dosages (15 and 20 mg/kg) it is effective against adult stages of flukes including *Dicrocoelium dendriticum* (92–98.9% efficacy) and *F. hepatica* (90.7% efficacy) [2]. Netobimin and its metabolites albendazole and albendazole oxide were all of low acute toxicity when given by oral route or parenteral routes. No deaths were caused with oral doses of up to 6900 mg/Kg body weight in mice, 5000 mg/Kg body weight in rats, and 2000 mg/Kg body weight in dogs. Intravenous LD_50_ values were 344 and 570 mg/Kg body weight in male and female mice, respectively, and 786 and 659 mg/Kg body weight in male and female rats, respectively [3].

Netobimin is not official in any pharmacopoeia; hence no official method is available for its estimation. Literature survey reveals different methods for the determination of netobimin in biological samples. Gokbulut et al. [4, 5] developed a method for the determination of netobimin in plasma, blood, and faecal excretions and its respective metabolites by high-performance liquid chromatography (HPLC) using a chiral phase-based HPLC following oral administration. Dowling et al. [6] developed an LC method with UV detection at 298 nm for the determination of netobimin in bovine liver. However, there are no reported methods for the determination of the drug in its pure powdered form or stability-indicating methods. This paper presents a study of alkaline degradation of netobimin, followed by the development of two chromatographic stability-indicating methods for the determination of the drug in its pure powder and in suspension forms.

The proposed HPLC and TLC densitometric methods are sensitive, fast, and stability indicating, used for simultaneous determination of netobimin in the presence of its degradation product. HPLC has the advantages of its discriminating power to resolve the drug from its degradation product and its ability to operate at ambient temperature that would not contribute to the degradation of the drug. TLC has become a routine analytical technique due to the possibility of application of several samples to be run simultaneously using a small amount of mobile phase, thus lowering the time and cost per analysis.

## 2. Experimental

### 2.1. Instruments

An Agilent HPLC instrument was obtained from Aligant (Waldbronn, Germany), and densitometer-dual wave length flying spot was obtained from Shimadzu (Tokyo, Japan), UV lamp with short wave length 254 nm was obtained from Desaga (Waldbronn, Germany), and thin layer chromatographic plates precoated with silica gel 60 F_254_ 10 × 20 cm were obtained from Fluka (Switzerland, Germany). The IR spectrophotometer was obtained from Bruker Optics (Ettlingen, Germany). Mass spectrophotometer was obtained from Agilent Technologies, Wilmington, DE. (Hewlett Packard Model 5988A GC/MS, fragmentation patterns arise from electron impact ionization.

### 2.2. Reagents

Methanol and acetonitrile HPLC grade were obtained from SD Fine-Chem Limited (Mumbai, India). Sodium hydroxide, hydrochloric acid, toluene, chloroform, methanol, ammonium hydroxide, and potassium dihydrogen phosphate were obtained from ADWIC (Cairo, Egypt).

### 2.3. Samples

#### 2.3.1. Reference Sample

 Netobimin-pure sample was kindly supplied by Shering-Plough Sante Animale La Grindoliere 49500 Serge, France B.N. 21787, its purity was found to be 99.9 ± 0.5% according to the manufacturer method [7].

#### 2.3.2. Pharmaceutical Formulation

 Hapadex oral suspension 5% was supplied by Shering-Plough Animale La Grindoliere 49500 Serge, France, B.N. 8389, each 1 mL is claimed to contain 50 mg of netobimin.

#### 2.3.3. Degraded Sample

The degradation processes under acid, alkaline, oxidative, and thermal conditions were followed by TLC using three different systems (toluene/methanol/chloroform/ammonium hydroxide (5 : 4 : 6 : 0.1 by volume), methanol: 33% ammonium hydroxide (9 : 0.1 v/v), and ethyl acetate: methanol: 33% ammonium hydroxide (8 : 1 : 0.2 by volume)), and the compound was found to be degraded under alkaline conditions, yielding a single component, as only one spot was obtained by TLC under the three different systems mentioned.

For the preparation of the alkaline degradation product, netobimin (50 mg) was refluxed with 50 mL 1 M NaOH solution for 5 hours and tested for complete degradation by TLC using toluene/methanol/chloroform/ammonium hydroxide (5 : 4 : 6 : 0.1 by volume) as the mobile phase. Only one spot was observed not corresponding to netobimin. The degraded solution was then cooled and neutralized with 2 M HCl till pH was approximately 7. The solution was nearly evaporated to dryness, cooled, and transferred quantitatively with methanol to prepare solution of concentration (equivalent to 0.5 mg/mL of intact netobimin) in methanol.

### 2.4. Standard Solutions

#### 2.4.1. Netobimin Stock Standard Solution

 (0.5 mg/mL) in methanol.

#### 2.4.2. Working Standard Solution

It is 0.05 mg/mL in mobile phase for method (A).

### 2.5. Degraded Solutions

#### 2.5.1. Degradation Product Stock Solution

 It is 0.5 mg/mL in methanol. Prepared as mentioned in Section 2.3.3.

#### 2.5.2. Working Degradation Product Solution

 It is 0.05 mg/mL in mobile phase for method (A).

### 2.6. Assessment of Selectivity/Specificity of the Methods

They are laboratory-prepared mixtures containing different ratios of netobimin and its degradation product.

#### 2.6.1. Method (A)

Aliquots (0.9–0.1 mL) of netobimin were accurately transferred from its working standard solution (0.05 mg/mL) equivalent to 45–5 *μ*g. Aliquots (0.1–0.9 mL) of working degradation product solution (0.05 mg/mL) equivalent to 5–45 *μ*g were added, to prepare solutions of concentration 9–1 *μ*g/mL of netobimin and 1–9 *μ*g/mL of the degradation product in the mobile phase containing 10–90% of the degradation product. 

#### 2.6.2. Method (B)

Aliquots (4.5–0.5 mL) of stock standard solution (0.5 mg/mL) equivalent to 2.25–0.25 mg were accurately transferred, then aliquots (0.5–4.5 mL) of degradation product stock solution (0.5 mg/mL) equivalent to 0.25–2.25 mg were added, to prepare solutions of concentration 450–50 *μ*g/mL of netobimin and 50–450 *μ*g/mL of the degradation product in methanol containing 10–90% of the degradation product.

### 2.7. Procedures

#### 2.7.1. Method (A)

**(i) Linearity**

Linearity was performed by preparing solutions of concentration range 1–10 *μ*g/mL in the mobile phase from the drug working standard solution (0.05 mg/mL). 20 *μ*L of the previously prepared solutions was injected in triplicate using the following chromatographic conditions.

- Column: nucleosil, size 125 × 4 mm, C18 5 *μ*m. The column was equilibrated with the mobile phase until steady baseline was obtained and column pressure was stabilized.
- Mobile phase: The mobile phase consisted of acetonitrile : methanol : 0.01 M potassium dihydrogen phosphate in ratio of 56 : 14 : 30 by volume. The mobile phase was filtered using 0.45 *μ*m membrane filters and degassed by ultrasonic vibrations for 30 min.
- Temperature: the system was operated at ambient temperature.
- Flow rate: the flow rate was isocratic at 0.5 mL/min.
- Detector wavelength: 254 nm.
- Injection volume: 20 *μ*L.

The chromatogram was obtained, the average peak area ratios obtained for each concentration of netobimin to that of external standard 1 *μ*g/mL were plotted versus concentrations, and the regression equation was computed.

**(ii) Accuracy**

The accuracy of the results was checked by applying the previously mentioned procedure under linearity for different concentrations of pure netobimin within the linearity range. The concentrations of the drug were calculated from the regression equation. The mean recovery percentage and relative standard deviation were then calculated.

**(iii) Precision**

**(a) Repeatability**

Three concentrations of netobimin stock standard solution (2, 4, and 6 *μ*g/mL) were analyzed three times each, intraday, using the previously mentioned procedure in Section 2.7.1(i). The mean recovery percentage and relative standard deviation were then calculated.

**(b) Intermediate Precision**

The previously mentioned netobimin samples were analyzed on three successive days using the procedure stated under Section 2.7.1(i). The mean recovery percentage and relative standard deviation were then calculated.

**(iv) Assessment of Selectivity/Specificity of the Method**

Twenty *μ*L from the prepared mixtures was injected into the liquid chromatograph. Then, the procedure was completed as described in Section 2.7.1(i). The concentration of netobimin was calculated by substitution in the corresponding regression equation.

**(v) Application of the Proposed Method for the Determination of Netobimin in Its Pharmaceutical Formulation**

The contents of the Hapadex oral suspension bottle were shacked well, then 1 mL equivalent to 50 mg netobimin was quantitatively transferred into a beaker, and 50 mL methanol was added. The beaker was covered with watch glass and the solution was stirred for 30 minutes using a magnetic stirrer, filtered, the residue was washed three times each with 10 mL methanol and filtered, and the collected filtrates were accurately transferred to prepare a solution of approximately 0.5 mg/mL in methanol. Suitable dilutions were made with the mobile phase to prepare a solution of approximately 4 *μ*g/mL in the mobile phase. Then the procedure was completed as described in Section 2.7.1(i). The concentration of netobimin was calculated by substitution in the corresponding regression equation.

**(vi) System Suitability**

The tailing factors, the resolution factor, the selectivity factor, the theoretical plate count, and the height equivalent to a theoretical plate (HETP) were calculated.

#### 2.7.2. Method (B)

**(i) Linearity**

Linearity was performed by preparing solutions of concentration range 50–500 *μ*g/mL in methanol from the stock standard solution (0.5 mg/mL). Ten *μ*L of the prepared solutions, using 10 *μ*L Hamilton syringe, was applied as separate compact bands 20 mm apart and 20 mm from the bottom of the plates. The chromatographic tank was saturated with the mobile phase for one hour in ascending manner to a distance of 7 cm from the spotting line at room temperature and air-dried, and the plates were scanned under the following conditions.

- Source of radiation: deuterium lamp.
- Photomode: reflection.
- Scan mode: zigzag.
- Result output: chromatogram and area under the peak.
- Swing width: 10 mm.
- Wavelength: 346 nm.

The scanning profile for netobimin was obtained. The calibration curve relating the integrated peak area to the corresponding concentration was constructed, and the regression equation was computed.

**(ii) Accuracy**

The accuracy of the results was checked by applying the proposed method for the determination of different concentrations of pure netobimin within the linearity range. The concentrations were calculated from the regression equation. The mean recovery percentage and relative standard deviation were then calculated.

**(iii) Precision**

**(a) Repeatability**

Three concentrations of netobimin stock standard solution (0.5, 1, and 2 *μ*g/band) were analyzed three times each, intraday, using the previously mentioned procedure under Section 2.7.2(i). The mean recovery percentage and relative standard deviation were then calculated.

**(b) Intermediate Precision**

The above-mentioned netobimin samples were analyzed on three successive days using the procedure stated in Section 2.7.2(i). The mean recovery percentage and relative standard deviation were then calculated.

**(iv) Assessment of Selectivity/Specificity of the Method**

Ten *μ*L of, the prepared mixtures was spotted on TLC plates and the procedure was completed as described in Section 2.7.2(i). The concentration of netobimin was calculated by substitution in the corresponding regression equation.

**(v) Application of the Proposed Method for the Determination of Netobimin in Its Pharmaceutical Formulation**

Four mL of the solution prepared (approximately 0.5 mg/mL) as described in Section 2.7.1(v) was accurately transferred to prepare a solution of approximately 0.2 mg/mL in methanol. Then the procedure was completed as described in Section 2.7.2(i). The concentration of netobimin was calculated by substitution in the corresponding regression equation.

## 3. Results and Discussion

The stability of netobimin was studied according to ICH guidelines [8] for the following.

- Acid and alkaline stress: 0.1 M HCl/0.1 M NaOH for 16 hrs, 0.2 M HCl/0.2 M NaOH for 16 hrs, and 1 M HCl/1 M NaOH for 4 hrs and 5 hrs.
- Oxidative condition: 3% H_2_O_2_ for 2 hrs, 4 hrs, 6 hrs, and 10 hrs.
- Thermal degradation: at 100°C in an oven for 2 hrs, 4 hrs, and 6 hrs.

**Figure 1:**
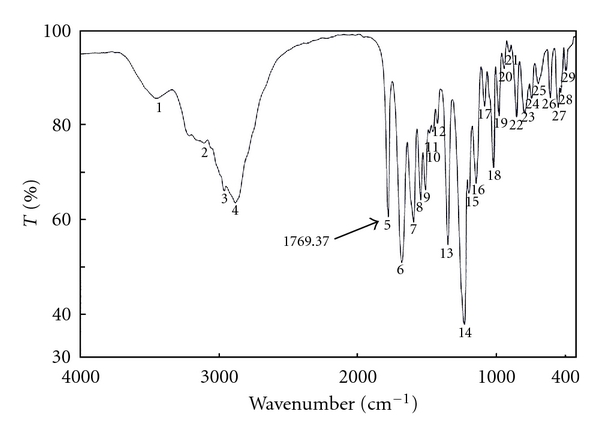
IR spectrum of intact netobimin.

**Figure 2:**
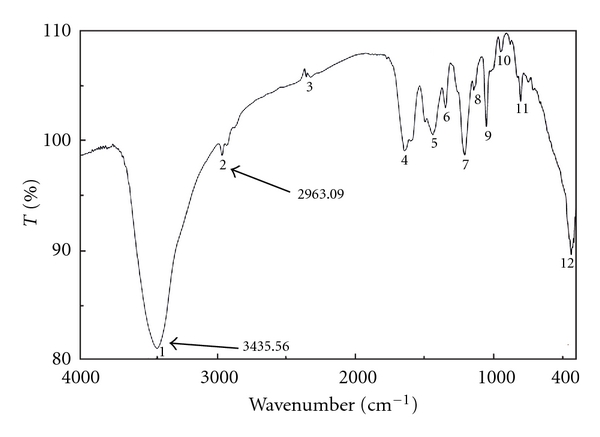
IR spectrum of the degradation product of netobimin.

**Figure 3:**
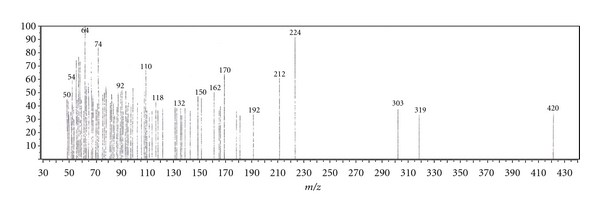
Mass spectrum of intact netobimin.

**Figure 4:**
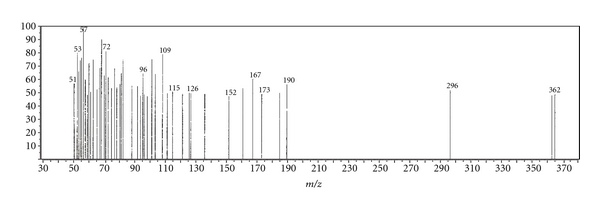
Mass spectrum of the degradation product of netobimin.

Since this work was concerned with the development of stability-indicating methods for the determination of netobimin, the degradation product was prepared in laboratory as mentioned in Section 2.3.3. The structure of the isolated alkaline degradation product was confirmed using IR and MS spectroscopy (Figures 1–4).

Figure 1 Shows the IR spectrum of the intact netobimin, which is characterized by the absorption frequency of C=O ester carbamate at 1769.37 cm^−1^. On the other hand the IR spectrum of the degradation product lacks the characteristic carbamate C=O band and it shows an NH_2_ functionality at 3435.56, 2963.09 cm^−1^ (Figure 2). This result is proved by using mass spectroscopy; the MS spectrum of the intact netobimin is characterized by the molecular ion *m/z* at 420 (M^+^., 35.56%) (Figure 3). On the other hand, the MS spectrum of the degradation product was characterized by the molecular ion *m/z* at 362 (M^+^., 48.07%) (Figure 4).

This finding suggested the degradation pathway and indicates that the degradation product of netobimin has the structure illustrated in Scheme 1.

### 3.1. Method (A)

HPLC has become the most versatile and widespread technique used by the pharmaceutical industry for quality control. It has many applications in the field of pharmaceuticals including the quantitative determination of drugs present either alone or in presence of their degradation [9–12].

The proposed method is based on the difference in the retention time between the intact drug and its degradation product. The suitable mobile phase has been selected to achieve the best separation of the drug from its degradation product.

**Figure 5:**
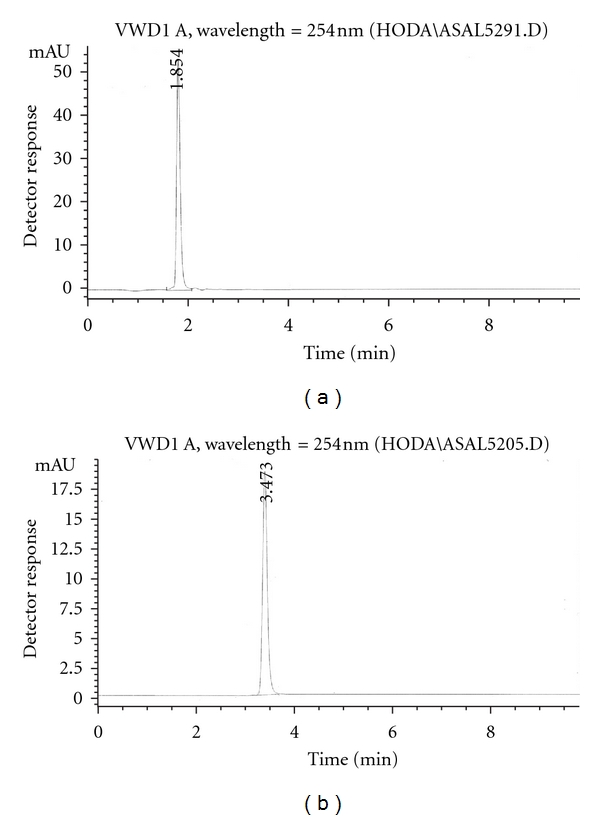
HPLC chromatogram of (a) the intact netobimin (7 *μ*g/mL) and (b) the degradation product (7 *μ*g/mL) using the specified chromatographic conditions.

Different mobile phases with different ratios were investigated such as methanol : 0.05 M potassium dihydrogen phosphate (60 : 40 v/v) and acetonitrile: 0.01 M potassium dihydrogen phosphate (70 : 30 v/v), but separation was not satisfactory. By using acetonitrile : methanol : 0.01 M potassium dihydrogen phosphate of 60 : 30 : 10 by volume, respectively, and of 40 : 30 : 30 by volume, respectively, poor resolution and tailed peaks were obtained, while upon adjusting the ratio to 56 : 14 : 30 by volume, respectively, satisfactory separation was performed, with a retention time of 1.834 ± 0.03 min for netobimin and 3.449 ± 0.03 min for its degradation product. This would permit quantitative determination of netobimin in presence of its alkaline-induced degradation product (Figure 5).

The average peak area ratios obtained for each concentration of netobimin to that of external standard 1 *μ*g/mL were plotted versus the corresponding concentration of the drug. The proposed method was found to be valid in the range of 1–10 *μ*g/mL, and the regression equation was computed and found to be

(1) A=0.9706C+0.0365,  r=0.9998,

where *A* is the peak area ratio, *C* is the concentration of the drug in *μ*g/mL, and *r* is the correlation coefficient.

The proposed method was successfully applied for the determination of the drug in pure powder form with mean percentage recovery of 99.3 ± 0.7% (Table 1).

System suitability test according to the United States Pharmacopoeia [13] was used to verify that the resolution and reproducibility of the chromatographic system were adequate for the analysis to be done. Accordingly, system suitability was checked by calculating the column efficiency (*N*), resolution (*R*), selectivity (*α*), and tailing factor (*T*), where the system was found to be suitable (Table 2).

### 3.2. Method (B)

Thin layer chromatography has become a well-established technique for the assay of drugs either in binary or in multicomponent mixtures [14, 15].

The proposed method is based on the difference in the *R*_*f*_ between the intact drug and its degradation product. The suitable mobile phase has been selected to achieve the best separation of the drug from its degradation product; other necessary conditions have been established.

**Figure 6:**
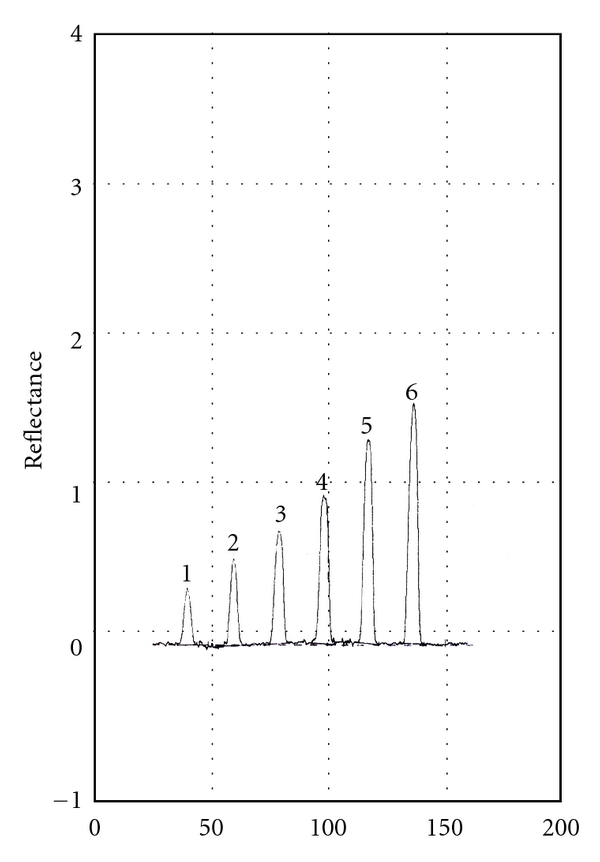
Scanning profile of TLC chromatogram of netobimin at 346 nm.

Different solvent systems with different ratios were tried including chloroform : acetone (4 : 1 v/v), where the drug and the degradation product have not retained. By using methanol : 33% ammonium hydroxide (9 : 0.1 v/v) separation of spots occurred but tailing was observed in the spot of the degradation. The use of toluene : methanol : chloroform : 33% ammonium hydroxide solution of 5 : 3 : 6 : 0.1 by volume resulted in poor separation, while upon adjusting the ratio to 5 : 4 : 6 : 0.1 by volume good separation of netobimin and its degradation product was obtained. The instrumental conditions for densitometric measurement such as scan mode and wavelength detection were optimized. The scan mode chosen was zigzag mode, and the wavelength was 346 nm. Netobimin was completely resolved from its degradation product, and its *R*_*f*_ value was 0.47. On the other hand, the *R*_*f*_ value of the degradation product was 0.74. This would permit quantitative determination of netobimin in presence of its alkaline-induced degradation product (Figure 6).

A linear relationship between the concentration of netobimin and the integrated peak area existed. The proposed method was found to be valid in the range of 0.5–5 *μ*g/band, and the regression equation was computed and found to be

(2) A=0.1086C+0.0684,  r=0.9994,

where *A* is the integrated peak area × 10^−4^, *C* is the concentration of the drug in *μ*g/band, and *r* is the correlation coefficient.

The proposed method was successfully applied for the determination of the drug in pure powder form with mean percentage recovery of 99.7 ± 0.7% (Table 1).

The specificity of the methods was proven by the analysis of laboratory-prepared mixtures containing different percentages of the degradation product. The proposed methods were found to be specific for netobimin in presence of up to 90% of its degradation product (Table 3).

The usefulness of the proposed methods for the analysis of netobimin was studied by assaying Hapadex oral suspension 5% (Table 4). Samples were also spiked in order to asses the validity of the proposed method (Table 4).

Results obtained by the proposed method for the determination of pure samples of the drug were statistically compared to those obtained by the manufacturer method of the drug [7], and no significant differences were observed (Table 5).

The accuracies were assessed by the determination of pure netobimin samples within the linearity ranges; the mean accuracies are given in (Table 1).

The repeatability and interday precision were evaluated by assaying three freshly prepared solutions of the drug in triplicate on the same day and on three successive days, respectively, at concentrations within the linearity range for both methods. RSD% shows the precision of the methods (Table 1).

Validation of the proposed methods was made by measuring range, accuracy, precision, repeatabilities, interday precision, linearity, and specificity. Results obtained are depicted in Table 1. This data render the applicability of the proposed method for the quality control of the drug formulation.

The proposed HPLC and TLC methods are precise, accurate, and sensitive. They can be used for the routine analysis of netobimin in pharmaceutical formulations and stability-indicating methods. The ICH guidelines were followed throughout the study for method validation.

## Figures and Tables

**Table 1:** Results of validation parameters of the responses and the regression equations obtained by the proposed methods.

Parameters	Method (A)	Method (B)
Slope^a^	0.9706	0.1086
Intercept^a^	0.0365	0.0684
Correlation coefficient	0.9998	0.9994
Concentration range	1–10 *μ*g/mL	0.5–5 *μ*g/band
Average accuracy (%)	99.3	99.7
S.D.	0.7	0.7
R.S.D.%	0.7	0.7
Specificity ± R.S.D.	99.4 ± 1.3	100.1 ± 1.2
Repeatability^b^ % ±R.S.D.	100.1 ± 0.1	99.8 ± 0.6
Intermediate precision^c^ % ±R.S.D.	99.8 ± 0.2	99.4 ± 0.2

^a^Results of five determinations.

^b^ *n* = 3 × 3.

^c^ *n* = 3 × 3.

**Table 2:** Parameters of system suitability test of method (A).

Parameter	Obtained value	
	Netobimin	Degradation product
Relative retention time (*α*)	1.88	
Resolution (*R*)	11.61	
Capacity factor (*K*)	1.22	3.18
Tailing factor (*T*)	0.8	0.86
Column efficiency (*N*)	4681	6551
HETP	0.0027 cm/plate	0.0019 cm/plate

**Table 3:** Results of analysis of netobimin in laboratory-prepared mixtures containing different ratios of netobimin and its degradation product in pure powder form by the proposed methods.

	Method (A)			Method (B)		
Degradation%	Concentration (*μ*g/mL)			Concentration (*μ*g/band)		
	Netobimin	Degradation product	Recovery %	Netobimin	Degradation product	Recovery %
10	9	1	100.8	4.5	0.5	98.1
20	8	2	98.2			
30	7	3	100.4	3.5	1.5	100.7
40	6	4	98.2			
50	5	5	100.8	2.5	2.5	100.9
60	4	6	98.2			
70	3	7	98.7	1.5	3.5	100.2
80	2	8	98.6			
90	1	9	100.8	0.5	4.5	100.5
Mean			99.4			100.1
S.D.			1.3			1.2
R.S.D. %			1.3			1.2

**Table 4:** Quantitative determination of netobimin in pharmaceutical formulation by the proposed methods and results of application of standard addition technique.

Hapadex oral suspension 5% B.N 8389	Method (A)			
	Found %^a^	Claimed amount taken (*μ*g/mL)	Standard added (*μ*g/mL)	Recovery %^b^ of added
	100.3	4	2	99.4
	98.9	4	4	99.0
	101.2	4	6	99.1
Mean	100.1			99.2
S.D.	1.2			0.2
R.S.D. %	1.2			0.2

Hapadex oral suspension 5% B.N 8389	Method (B)			
	Found %^a^	Claimed amount taken (*μ*g/band)	Standard added (*μ*g/band)	Recovery %^b^ of added

	100.5	2	1	99.3
	100.5	2	2	99.4
	99.7	2	3	100.7
Mean	100.2			99.8
S.D.	0.5			0.8
R.S.D.%	0.5			0.8

^a^Average of six determinations.

^b^Average of six determinations.

**Table 5:** Statistical analysis of the results obtained for the determination of netobimin in pure samples by the proposed methods and those obtained by the manufacturer method.

Item	Method (A)	Method (B)	Manufacturer's method *[7]
Mean	99.4	99.7	99.9
S.D.	0.7	0.7	0.5
R.S.D %	0.7	0.7	0.5
Variance	0.5	0.5	0.3
*n*	10	6	5
Student*ۥ*s *t*	1.4 (2.160)	0.5 (2.262)	
*F* test	1.7 (4.77)	1.7 (5.05)	

Figures in parentheses are the corresponding tabulated values at *P* = 0.05.

*[7] Manufacturer method HPLC, mobile phase methanol : 0.05 M monobasic potassium phosphate of 60 : 40 by volume, respectively, UV set at 254 nm.
